# (*E*)-2-(Furan-2-yl­methyl­idene)-8-methyl-2,3,4,9-tetra­hydro-1*H*-carbazol-1-one

**DOI:** 10.1107/S1600536810045599

**Published:** 2010-11-13

**Authors:** R. Archana, E. Yamuna, K. J. Rajendra Prasad, A. Thiruvalluvar, R. J. Butcher

**Affiliations:** a PG Research Department of Physics, Rajah Serfoji Government College (Autonomous), Thanjavur 613 005, Tamilnadu, India; b Department of Chemistry, Bharathiar University, Coimbatore 641 046, Tamilnadu, India; c Department of Chemistry, Howard University, 525 College Street NW, Washington, DC 20059, USA

## Abstract

In the title mol­ecule, C_18_H_15_NO_2_, the carbazole unit is not planar [maximum deviation from mean plane = 0.236 (2) Å]. The pyrrole ring makes dihedral angles of 1.21 (10) and 16.74 (12)° with the benzene and the furan rings, respectively. The cyclo­hexene ring adopts a half-chair conformation. In the crystal, inversion dimers linked by pairs of N—H⋯O hydrogen bonds generate *R*_2_^2^(10) loops.

## Related literature

For the synthesis of hetero-annulated carbazoles, see: Knölker & Reddy (2002 ▶). For the derivation of various hetero-annulated carbazoles, see: Sridharan *et al.* (2008 ▶); Danish & Rajendra Prasad (2004 ▶, 2005 ▶); Periyasami *et al.* (2008 ▶, 2009 ▶). For ring conformations, see: Cremer & Pople (1975 ▶). For hydrogen-bond motifs, see: Bernstein *et al.* (1995 ▶).
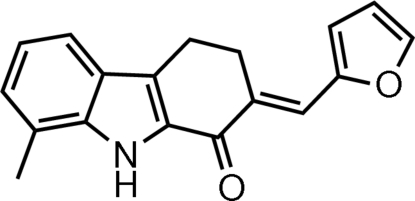


## Experimental

### 

#### Crystal data

C_18_H_15_NO_2_
*M* *_r_* = 277.31
Orthorhombic, 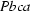

*a* = 6.7353 (1) Å
*b* = 16.1393 (3) Å
*c* = 25.9549 (4) Å
*V* = 2821.38 (8) Å^3^
*Z* = 8
Cu *K*α radiation
μ = 0.68 mm^−1^
*T* = 295 K
0.44 × 0.28 × 0.12 mm

#### Data collection

Oxford Diffraction Xcalibur Ruby Gemini diffractometer
Absorption correction: multi-scan (*CrysAlis PRO*; Oxford Diffraction, 2010 ▶) *T*_min_ = 0.888, *T*_max_ = 1.000
6361 measured reflections
2736 independent reflections
2201 reflections with *I* > 2σ(*I*)
*R*_int_ = 0.026

#### Refinement

*R*[*F* ^2^ > 2σ(*F* ^2^)] = 0.050
*wR*(*F* ^2^) = 0.157
*S* = 1.05
2736 reflections
195 parameters
H atoms treated by a mixture of independent and constrained refinement
Δρ_max_ = 0.23 e Å^−3^
Δρ_min_ = −0.26 e Å^−3^

### 

Data collection: *CrysAlis PRO* (Oxford Diffraction, 2010 ▶); cell refinement: *CrysAlis PRO*; data reduction: *CrysAlis PRO*; program(s) used to solve structure: *SHELXS97* (Sheldrick, 2008 ▶); program(s) used to refine structure: *SHELXL97* (Sheldrick, 2008 ▶); molecular graphics: *ORTEP-3* (Farrugia, 1997 ▶) and *PLATON* (Spek, 2009 ▶); software used to prepare material for publication: *PLATON*.

## Supplementary Material

Crystal structure: contains datablocks global, I. DOI: 10.1107/S1600536810045599/si2306sup1.cif

Structure factors: contains datablocks I. DOI: 10.1107/S1600536810045599/si2306Isup2.hkl

Additional supplementary materials:  crystallographic information; 3D view; checkCIF report

## Figures and Tables

**Table 1:** Hydrogen-bond geometry (Å, °)

*D*—H⋯*A*	*D*—H	H⋯*A*	*D*⋯*A*	*D*—H⋯*A*
N9—H9⋯O1^i^	0.89 (3)	2.01 (3)	2.8969 (19)	176 (2)

Symmetry code: (i) 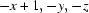.

**Fig. 1.**
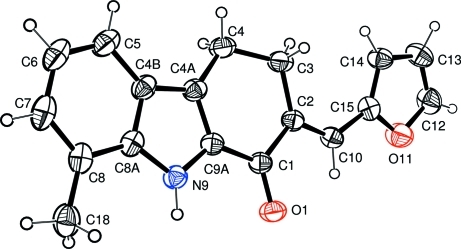
The molecular structure of the title compound, showing the atom-numbering scheme and displacement ellipsoids drawn at the 30% probability level. H atoms are shown as small spheres of arbitrary radius.

**Fig. 2.**
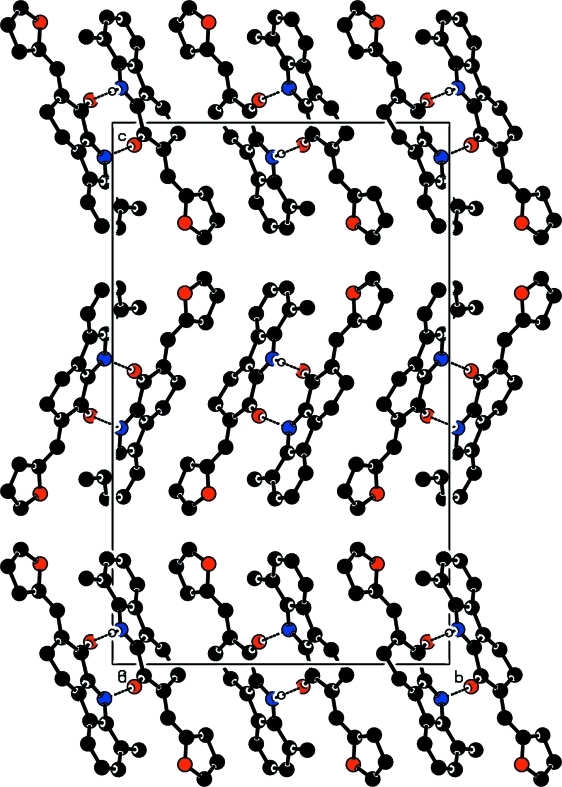
The molecular packing of the title compound, viewed down the a axis. Dashed lines indicate hydrogen bonds. H atoms not involved in hydrogen bonding have been omitted.

## supplementary crystallographic information

### Comment

As a result of their significant potential as therapeutics, interest has grown in the development of methods for the efficient and rapid synthesis of the derivatives of various hetero-annulated carbazoles especially because the current methods, which involve multi-step reactions, lower yields, longer reaction times, and high cost of palladium (Knölker & Reddy (2002)) are unsatisfactory. Herein, therefore, we report the easily accessible synthon (*E*)-2-(furan-2-yl methylene)-8-methyl-2,3,4,9-tetrahydro-1*H*-carbazol-1-one to derive various hetero-annulated carbazoles (Sridharan *et al.*, (2008); Danish & Rajendra Prasad (2004, 2005); Periyasami *et al.*, (2008, 2009)).

In the title molecule (Scheme I, Fig. 1), C_18_H_15_NO_2_, the carbazole unit is not planar. The pyrrole ring makes dihedral angles of 1.21 (10)° and 16.74 (12)° with the benzene and the furan rings respectively. The cyclohexene ring adopts a half-chair conformation. The puckering parameters (Cremer & Pople, 1975) are q2=0.232 (2) Å, q3=-0.153 (2) Å, Q=0.278 (2) Å, θ=123.4 (4)° and φ=322.6 (5)°. Intermolecular N9—H9···O1 hydrogen bonds form a *R*_2_^2^(10) (Bernstein *et al.*, 1995) ring in the crystal structure (Table 1, Fig. 2).

### Experimental

An equimolar mixture of 8-methyl-2,3,4,9-tetrahydro-1*H*-carbazol-1-one (0.995 g, 0.005 mol) and furan-2-carbaldehyde (0.41 ml, 0.005 mol) was treated with 25 ml of a 5% ethanolic potassium hydroxide solution and stirred for 6 h at room temperature. The product precipitated as a yellow crystalline mass, was filtered off and washed with 50% ethanol. A further crop of condensation product was obtained on neutralization with acetic acid and dilution with water. The product was recrystallized from methanol to yield 90% (1.246 g) of the title compound. The pure compound was recrystallized from EtOAc.

### Refinement

The H atom bonded to N9 was located in a difference Fourier map and refined freely. Other H atoms were positioned geometrically and allowed to ride on their parent atoms, with C—H = 0.93–0.97 Å and *U*_iso_(H) = 1.2–1.5*U*_eq_(parent atom).

### Crystal data

**Table:**

C_18_H_15_NO_2_	*D*_x_ = 1.306 Mg m^−^^3^
*M**_r_* = 277.31	Melting point: 505 K
Orthorhombic, *P**b**c**a*	Cu *K*α radiation, λ = 1.54184 Å
Hall symbol: -P 2ac 2ab	Cell parameters from 3160 reflections
*a* = 6.7353 (1) Å	θ = 4.4–72.5°
*b* = 16.1393 (3) Å	µ = 0.68 mm^−^^1^
*c* = 25.9549 (4) Å	*T* = 295 K
*V* = 2821.38 (8) Å^3^	Prism, pale-yellow
*Z* = 8	0.44 × 0.28 × 0.12 mm
*F*(000) = 1168	

### Data collection

**Table:**

Oxford Diffraction Xcalibur Ruby Gemini diffractometer	2736 independent reflections
Radiation source: Enhance (Cu) X-ray Source	2201 reflections with *I* > 2σ(*I*)
graphite	*R*_int_ = 0.026
Detector resolution: 10.5081 pixels mm^-1^	θ_max_ = 72.7°, θ_min_ = 5.7°
ω scans	*h* = −5→8
Absorption correction: multi-scan (*CrysAlis PRO*; Oxford Diffraction, 2010)	*k* = −19→13
*T*_min_ = 0.888, *T*_max_ = 1.000	*l* = −31→31
6361 measured reflections	

### Refinement

**Table:**

Refinement on *F*^2^	Primary atom site location: structure-invariant direct methods
Least-squares matrix: full	Secondary atom site location: difference Fourier map
*R*[*F*^2^ > 2σ(*F*^2^)] = 0.050	Hydrogen site location: inferred from neighbouring sites
*wR*(*F*^2^) = 0.157	H atoms treated by a mixture of independent and constrained refinement
*S* = 1.05	*w* = 1/[σ^2^(*F*_o_^2^) + (0.0921*P*)^2^ + 0.408*P*] where *P* = (*F*_o_^2^ + 2*F*_c_^2^)/3
2736 reflections	(Δ/σ)_max_ = 0.001
195 parameters	Δρ_max_ = 0.23 e Å^−^^3^
0 restraints	Δρ_min_ = −0.26 e Å^−^^3^

### Special details

**Table:**

Geometry. Bond distances, angles *etc*. have been calculated using the rounded fractional coordinates. All su's are estimated from the variances of the (full) variance-covariance matrix. The cell e.s.d.'s are taken into account in the estimation of distances, angles and torsion angles
Refinement. Refinement of *F*^2^ against ALL reflections. The weighted *R*-factor *wR* and goodness of fit *S* are based on *F*^2^, conventional *R*-factors *R* are based on *F*, with *F* set to zero for negative *F*^2^. The threshold expression of *F*^2^ > 2σ(*F*^2^) is used only for calculating *R*-factors(gt) *etc*. and is not relevant to the choice of reflections for refinement. *R*-factors based on *F*^2^ are statistically about twice as large as those based on *F*, and *R*- factors based on ALL data will be even larger.

### Fractional atomic coordinates and isotropic or equivalent isotropic displacement parameters (Å^2^)

**Table:**

	*x*	*y*	*z*	*U*_iso_*/*U*_eq_	
O1	0.3691 (2)	0.06478 (9)	−0.04131 (5)	0.0638 (5)	
O11	0.0871 (3)	0.21485 (11)	−0.18520 (6)	0.0810 (6)	
N9	0.2678 (2)	0.02422 (9)	0.06403 (5)	0.0489 (4)	
C1	0.2176 (3)	0.09332 (11)	−0.02148 (6)	0.0475 (5)	
C2	0.0821 (2)	0.15134 (10)	−0.04916 (7)	0.0473 (5)	
C3	−0.0829 (3)	0.19426 (12)	−0.01982 (8)	0.0605 (6)	
C4	−0.1653 (3)	0.14925 (13)	0.02677 (8)	0.0615 (6)	
C4A	−0.0102 (3)	0.10045 (11)	0.05422 (7)	0.0508 (5)	
C4B	−0.0102 (3)	0.06700 (11)	0.10466 (7)	0.0537 (6)	
C5	−0.1417 (3)	0.07341 (14)	0.14698 (8)	0.0679 (7)	
C6	−0.0955 (4)	0.03192 (16)	0.19083 (9)	0.0735 (8)	
C7	0.0762 (3)	−0.01716 (14)	0.19432 (8)	0.0681 (7)	
C8	0.2108 (3)	−0.02535 (12)	0.15467 (7)	0.0561 (6)	
C8A	0.1646 (3)	0.01922 (10)	0.10952 (6)	0.0497 (5)	
C9A	0.1612 (3)	0.07363 (10)	0.03084 (6)	0.0466 (5)	
C10	0.1218 (3)	0.16433 (11)	−0.09919 (7)	0.0518 (5)	
C12	−0.0226 (4)	0.27239 (15)	−0.21183 (9)	0.0775 (8)	
C13	−0.1434 (4)	0.31065 (15)	−0.18050 (9)	0.0736 (8)	
C14	−0.1132 (3)	0.27681 (12)	−0.13083 (7)	0.0586 (6)	
C15	0.0280 (3)	0.21920 (11)	−0.13515 (7)	0.0551 (6)	
C18	0.3917 (3)	−0.07814 (15)	0.15847 (8)	0.0718 (8)	
H3A	−0.19153	0.20433	−0.04355	0.0725*	
H3B	−0.03417	0.24784	−0.00856	0.0725*	
H4A	−0.22223	0.18937	0.05035	0.0738*	
H4B	−0.27067	0.11228	0.01579	0.0738*	
H5	−0.25654	0.10519	0.14480	0.0815*	
H6	−0.17930	0.03607	0.21919	0.0882*	
H7	0.10015	−0.04552	0.22489	0.0818*	
H9	0.382 (4)	−0.0012 (13)	0.0577 (9)	0.063 (6)*	
H10	0.22576	0.13304	−0.11252	0.0622*	
H12	−0.01222	0.28261	−0.24699	0.0931*	
H13	−0.23267	0.35233	−0.18921	0.0884*	
H14	−0.17937	0.29187	−0.10080	0.0704*	
H18A	0.39152	−0.10687	0.19085	0.1077*	
H18B	0.50785	−0.04389	0.15617	0.1077*	
H18C	0.39214	−0.11766	0.13083	0.1077*	

### Atomic displacement parameters (Å^2^)

**Table:**

	*U*^11^	*U*^22^	*U*^33^	*U*^12^	*U*^13^	*U*^23^
O1	0.0598 (8)	0.0799 (9)	0.0517 (7)	0.0239 (7)	0.0087 (6)	0.0061 (6)
O11	0.0930 (12)	0.0872 (11)	0.0627 (9)	0.0242 (9)	0.0049 (8)	0.0038 (8)
N9	0.0503 (8)	0.0537 (8)	0.0428 (7)	0.0025 (7)	0.0021 (6)	−0.0015 (6)
C1	0.0486 (9)	0.0478 (9)	0.0460 (9)	0.0025 (7)	0.0006 (7)	−0.0052 (7)
C2	0.0471 (9)	0.0464 (8)	0.0485 (9)	0.0012 (7)	−0.0018 (7)	−0.0039 (7)
C3	0.0562 (10)	0.0619 (11)	0.0633 (12)	0.0158 (9)	0.0043 (9)	0.0010 (9)
C4	0.0534 (10)	0.0664 (11)	0.0647 (12)	0.0118 (9)	0.0092 (9)	−0.0047 (9)
C4A	0.0500 (9)	0.0496 (9)	0.0528 (10)	−0.0019 (7)	0.0042 (7)	−0.0084 (8)
C4B	0.0550 (10)	0.0557 (10)	0.0503 (10)	−0.0077 (8)	0.0080 (8)	−0.0092 (8)
C5	0.0656 (12)	0.0755 (13)	0.0627 (12)	−0.0084 (10)	0.0200 (10)	−0.0104 (10)
C6	0.0797 (15)	0.0856 (15)	0.0553 (12)	−0.0232 (12)	0.0225 (11)	−0.0094 (11)
C7	0.0816 (15)	0.0771 (13)	0.0457 (10)	−0.0289 (12)	0.0024 (10)	0.0011 (9)
C8	0.0672 (11)	0.0566 (10)	0.0444 (9)	−0.0180 (9)	−0.0046 (8)	−0.0018 (8)
C8A	0.0546 (9)	0.0514 (9)	0.0431 (9)	−0.0118 (8)	0.0025 (7)	−0.0072 (7)
C9A	0.0491 (8)	0.0462 (8)	0.0446 (9)	0.0009 (7)	−0.0007 (7)	−0.0055 (7)
C10	0.0548 (9)	0.0478 (9)	0.0529 (10)	0.0049 (8)	−0.0025 (8)	−0.0045 (7)
C12	0.0962 (16)	0.0823 (14)	0.0541 (12)	0.0093 (13)	−0.0067 (12)	0.0136 (11)
C13	0.0794 (14)	0.0662 (13)	0.0753 (15)	0.0177 (11)	−0.0178 (12)	0.0001 (11)
C14	0.0643 (11)	0.0643 (11)	0.0473 (10)	0.0131 (9)	−0.0024 (8)	−0.0073 (8)
C15	0.0623 (10)	0.0526 (9)	0.0505 (10)	−0.0036 (8)	−0.0064 (8)	−0.0008 (8)
C18	0.0778 (14)	0.0819 (15)	0.0556 (12)	−0.0085 (12)	−0.0087 (10)	0.0123 (10)

### Geometric parameters (Å, °)

**Table:**

O1—C1	1.232 (2)	C8—C8A	1.410 (2)
O11—C12	1.373 (3)	C10—C15	1.433 (3)
O11—C15	1.361 (2)	C12—C13	1.306 (4)
N9—C8A	1.373 (2)	C13—C14	1.415 (3)
N9—C9A	1.376 (2)	C14—C15	1.335 (3)
N9—H9	0.89 (3)	C3—H3A	0.9700
C1—C2	1.492 (2)	C3—H3B	0.9700
C1—C9A	1.446 (2)	C4—H4A	0.9700
C2—C3	1.515 (3)	C4—H4B	0.9700
C2—C10	1.342 (3)	C5—H5	0.9300
C3—C4	1.516 (3)	C6—H6	0.9300
C4—C4A	1.490 (3)	C7—H7	0.9300
C4A—C9A	1.374 (3)	C10—H10	0.9300
C4A—C4B	1.416 (3)	C12—H12	0.9300
C4B—C5	1.415 (3)	C13—H13	0.9300
C4B—C8A	1.413 (3)	C14—H14	0.9300
C5—C6	1.357 (3)	C18—H18A	0.9600
C6—C7	1.405 (3)	C18—H18B	0.9600
C7—C8	1.378 (3)	C18—H18C	0.9600
C8—C18	1.490 (3)		
			
O1···N9	2.8930 (19)	C14···H5^iii^	3.0900
O1···N9^i^	2.8969 (19)	C14···H3A	2.6000
O11···C7^ii^	3.383 (3)	C15···H3A	2.8100
O1···H9	2.78 (2)	C18···H9	2.90 (2)
O1···H9^i^	2.01 (3)	C18···H10^i^	2.9700
O1···H10	2.3600	H3A···C14	2.6000
N9···O1	2.8930 (19)	H3A···C15	2.8100
N9···O1^i^	2.8969 (19)	H3A···H14	2.0500
C1···C4A^ii^	3.529 (3)	H3B···C4^iii^	3.0300
C2···C8A^ii^	3.577 (2)	H3B···H4A^iii^	2.5700
C3···C14	3.181 (3)	H4A···C2^iv^	2.8900
C4A···C1^ii^	3.529 (3)	H4A···C10^iv^	2.8800
C7···O11^ii^	3.383 (3)	H4A···H3B^iv^	2.5700
C8···C15^ii^	3.554 (3)	H5···C13^iv^	3.0800
C8···C10^ii^	3.482 (3)	H5···C14^iv^	3.0900
C8A···C10^ii^	3.545 (3)	H6···C7^vii^	2.9100
C8A···C2^ii^	3.577 (2)	H6···H7^vii^	2.4600
C8A···C13^iii^	3.550 (3)	H7···H18A	2.3700
C9A···C9A^ii^	3.595 (2)	H7···H6^vi^	2.4600
C10···C8A^ii^	3.545 (3)	H9···O1	2.78 (2)
C10···C8^ii^	3.482 (3)	H9···C18	2.90 (2)
C13···C8A^iv^	3.550 (3)	H9···O1^i^	2.01 (3)
C14···C3	3.181 (3)	H10···O1	2.3600
C15···C8^ii^	3.554 (3)	H10···C18^i^	2.9700
C2···H4A^iii^	2.8900	H10···H18B^i^	2.5600
C3···H14	2.7100	H13···C6^iv^	3.0800
C4···H3B^iv^	3.0300	H13···C7^iv^	2.9600
C4A···H14^iii^	3.0700	H13···C8^iv^	2.9600
C5···H18B^v^	3.0400	H13···C8A^iv^	3.0100
C6···H18B^v^	3.0700	H14···C3	2.7100
C6···H13^iii^	3.0800	H14···H3A	2.0500
C7···H6^vi^	2.9100	H14···C4A^iv^	3.0700
C7···H13^iii^	2.9600	H14···C9A^iv^	3.0300
C8···H13^iii^	2.9600	H18A···H7	2.3700
C8A···H13^iii^	3.0100	H18B···C5^viii^	3.0400
C9A···H14^iii^	3.0300	H18B···C6^viii^	3.0700
C10···H4A^iii^	2.8800	H18B···H10^i^	2.5600
C13···H5^iii^	3.0800		
			
C12—O11—C15	106.75 (18)	C10—C15—C14	133.61 (18)
C8A—N9—C9A	107.96 (14)	O11—C15—C10	117.45 (17)
C9A—N9—H9	127.2 (15)	O11—C15—C14	108.94 (17)
C8A—N9—H9	124.8 (15)	C2—C3—H3A	108.00
O1—C1—C9A	121.86 (17)	C2—C3—H3B	108.00
O1—C1—C2	122.69 (15)	C4—C3—H3A	108.00
C2—C1—C9A	115.44 (16)	C4—C3—H3B	108.00
C3—C2—C10	124.13 (16)	H3A—C3—H3B	107.00
C1—C2—C3	119.58 (16)	C3—C4—H4A	109.00
C1—C2—C10	116.23 (15)	C3—C4—H4B	109.00
C2—C3—C4	116.77 (16)	C4A—C4—H4A	109.00
C3—C4—C4A	112.22 (17)	C4A—C4—H4B	109.00
C4B—C4A—C9A	106.75 (16)	H4A—C4—H4B	108.00
C4—C4A—C4B	130.07 (18)	C4B—C5—H5	121.00
C4—C4A—C9A	122.99 (16)	C6—C5—H5	121.00
C5—C4B—C8A	119.49 (17)	C5—C6—H6	119.00
C4A—C4B—C5	133.62 (19)	C7—C6—H6	119.00
C4A—C4B—C8A	106.89 (16)	C6—C7—H7	118.00
C4B—C5—C6	118.1 (2)	C8—C7—H7	118.00
C5—C6—C7	121.4 (2)	C2—C10—H10	115.00
C6—C7—C8	123.2 (2)	C15—C10—H10	115.00
C7—C8—C8A	115.26 (18)	O11—C12—H12	125.00
C8A—C8—C18	121.82 (17)	C13—C12—H12	125.00
C7—C8—C18	122.92 (18)	C12—C13—H13	126.00
N9—C8A—C8	129.29 (17)	C14—C13—H13	126.00
C4B—C8A—C8	122.45 (17)	C13—C14—H14	126.00
N9—C8A—C4B	108.25 (14)	C15—C14—H14	126.00
C1—C9A—C4A	124.50 (17)	C8—C18—H18A	109.00
N9—C9A—C1	125.34 (17)	C8—C18—H18B	109.00
N9—C9A—C4A	110.15 (14)	C8—C18—H18C	109.00
C2—C10—C15	129.69 (17)	H18A—C18—H18B	109.00
O11—C12—C13	110.0 (2)	H18A—C18—H18C	109.00
C12—C13—C14	107.2 (2)	H18B—C18—H18C	109.00
C13—C14—C15	107.15 (18)		
			
C15—O11—C12—C13	0.0 (3)	C9A—C4A—C4B—C8A	−0.8 (2)
C12—O11—C15—C10	179.89 (19)	C4—C4A—C9A—N9	−174.75 (17)
C12—O11—C15—C14	0.2 (2)	C4—C4A—C9A—C1	4.0 (3)
C9A—N9—C8A—C4B	−0.28 (19)	C4B—C4A—C9A—N9	0.6 (2)
C9A—N9—C8A—C8	178.49 (18)	C4B—C4A—C9A—C1	179.32 (17)
C8A—N9—C9A—C1	−178.91 (17)	C4A—C4B—C5—C6	179.8 (2)
C8A—N9—C9A—C4A	−0.2 (2)	C8A—C4B—C5—C6	−1.0 (3)
O1—C1—C2—C3	−170.47 (17)	C4A—C4B—C8A—N9	0.7 (2)
O1—C1—C2—C10	7.0 (3)	C4A—C4B—C8A—C8	−178.22 (17)
C9A—C1—C2—C3	8.5 (2)	C5—C4B—C8A—N9	−178.77 (17)
C9A—C1—C2—C10	−174.06 (16)	C5—C4B—C8A—C8	2.4 (3)
O1—C1—C9A—N9	1.6 (3)	C4B—C5—C6—C7	−0.9 (3)
O1—C1—C9A—C4A	−176.90 (18)	C5—C6—C7—C8	1.5 (4)
C2—C1—C9A—N9	−177.39 (15)	C6—C7—C8—C8A	−0.1 (3)
C2—C1—C9A—C4A	4.1 (3)	C6—C7—C8—C18	−179.4 (2)
C1—C2—C3—C4	−28.3 (2)	C7—C8—C8A—N9	179.62 (18)
C10—C2—C3—C4	154.45 (18)	C7—C8—C8A—C4B	−1.8 (3)
C1—C2—C10—C15	−176.11 (18)	C18—C8—C8A—N9	−1.1 (3)
C3—C2—C10—C15	1.2 (3)	C18—C8—C8A—C4B	177.53 (18)
C2—C3—C4—C4A	33.9 (2)	C2—C10—C15—O11	−174.38 (19)
C3—C4—C4A—C4B	162.79 (19)	C2—C10—C15—C14	5.2 (4)
C3—C4—C4A—C9A	−23.0 (3)	O11—C12—C13—C14	−0.2 (3)
C4—C4A—C4B—C5	−6.5 (4)	C12—C13—C14—C15	0.4 (3)
C4—C4A—C4B—C8A	174.15 (19)	C13—C14—C15—O11	−0.4 (2)
C9A—C4A—C4B—C5	178.5 (2)	C13—C14—C15—C10	−180.0 (2)

Symmetry codes: (i) −*x*+1, −*y*, −*z*; (ii) −*x*, −*y*, −*z*; (iii) *x*+1/2, −*y*+1/2, −*z*; (iv) *x*−1/2, −*y*+1/2, −*z*; (v) *x*−1, *y*, *z*; (vi) *x*+1/2, *y*, −*z*+1/2; (vii) *x*−1/2, *y*, −*z*+1/2; (viii) *x*+1, *y*, *z*.

### Hydrogen-bond geometry (Å, °)

**Table:**

*D*—H···*A*	*D*—H	H···*A*	*D*···*A*	*D*—H···*A*
N9—H9···O1^i^	0.89 (3)	2.01 (3)	2.8969 (19)	176 (2)

Symmetry codes: (i) −*x*+1, −*y*, −*z*.
